# Serological survey of *Leishmania*infection in blood donors in Salvador, Northeastern Brazil

**DOI:** 10.1186/1471-2334-14-422

**Published:** 2014-07-30

**Authors:** Kiyoshi F Fukutani, Virgínia Figueiredo, Fabiana S Celes, Juqueline R Cristal, Aldina Barral, Manoel Barral-Netto, Camila I de Oliveira

**Affiliations:** Centro de Pesquisas Gonçalo Moniz, Fundação Oswaldo Cruz (FIOCRUZ), Rua Waldemar Falcão, 121, Salvador, BA Brazil; Fundação de Hematologia e Hemoterapia da Bahia (HEMOBA/SESAB), Av. Vasco da Gama, s/n, Salvador, BA Brazil; Instituto de Investigação em Imunologia, Salvador, BA Brazil

**Keywords:** *Leishmania*, Blood supply, Asymptomatic infection, PCR, ELISA

## Abstract

**Background:**

Visceral Leishmaniasis is endemic to Brazil, where it is caused by *Leishmania infantum* (syn. *Leishmania chagasi*). Following parasite inoculation, individuals may experience asymptomatic infection, raising the possibility of parasite transmission through the transfusion of contaminated blood products. In the present work, we evaluated the prevalence of asymptomatic *Leishmania* infection among blood donors in Salvador, northeastern Brazil.

**Methods:**

Peripheral blood was collected from 700 blood donors attending the Blood Bank of Bahia (HEMOBA/SESAB), from January to September 2010. We evaluated anti-*Leishmania* serology by ELISA, employing Soluble *Leishmania* Antigen (sensitivity 90% and specificity 95%). The presence of parasite DNA was determined by qPCR, targeting a single copy gene (G6PD), and by end-point PCR, targeting multiple targets, namely a segment located in the *Leishmania* rRNA locus (ITS) and the conserved region of kinetoplastid DNA (kDNA) minicircles.

**Results:**

The blood-donor population was comprised of 74.5% of males with a mean age of 34 years. Anti-*Leishmania* serology by ELISA was positive in 5.4% (38/700) individuals. One individual was also positive for Chagas’ disease and another tested positive for Syphilis. Employing qPCR, parasite DNA was not found in any of 38 seropositive samples. However, by ITS PCR, 8/38 (21%) samples were positive and this positivity increased to 26/38 (68%) when we targeted kDNA amplification. Agreement between both techniques (ITS and kDNA PCR) was fair (*kappa* = 0.219).

**Conclusions:**

These results indicate that asymptomatic infection is present among the blood donor population of Salvador, a finding that warrants a broader discussion regarding the need to implement specific screening strategies.

**Electronic supplementary material:**

The online version of this article (doi:10.1186/1471-2334-14-422) contains supplementary material, which is available to authorized users.

## Background

Visceral leishmaniasis (VL) or kalazar is a serious public health problem worldwide, and approximately 500,000 new cases are reported each year {[1] #67}. *Leishmania infantum* is the causative agent of VL in Brazil, which accounts for 90% of cases in the Americas {[2] #257} and where 3392 VL cases were reported in 2012 {[3], #258}. In the last decades, VL is becoming more prevalent in urban centers of Brazil, especially in expanding cities with different patterns of economic and social development {[4] #259}. Patent VL is classically characterized by the presence of irregular fever, paleness, and splenomegaly and, in the absence of treatment; it is a progressive infection with fatal outcome {[3] #279}. However, a large number of infections remain asymptomatic, in which individuals are infected by *Leishmania* but display an apparent healthy condition (rev. in {[5] #260}). There are concerns that asymptomatic individuals remain carriers of the infection and, as such, may play an important role in the epidemiology of VL, especially in urban concentrations where the disease is present {[6] #261}.

Classically, transmission of *Leishmania* parasites occurs through sand fly bites, however, studies in France {[7] #248}, Italy {[8] #6}, Spain {[9] #12}, India {[10] #9}, Bangladesh {[11] #250} and Turkey {[12] #251} showed that asymptomatic individuals are found among blood donors, suggesting that transfusion of *Leishmania*-contaminated blood products might be a source of disease transmission in parallel to syringe sharing {[13] #68} and organ transplantation {[14] #7; [15] #8; [16] #19; [17] #41; [18] #246}. In northeastern Brazil, the prevalence of anti-*Leishmania* antibodies was 9% among blood donors, increased to 25% in a VL focus and was highest (37%) among poly-transfused hemodialysis patients {[19] #37}. In a subsequent study, upon evaluation of 21 sero-positive blood-donors, authors found that five individuals (23%) were positive to *Leishmania* by PCR whereas dot-blot hybridization increased this positivity to nine individuals (43%) {[20] #10}. In central western Brazil, the prevalence of anti-*Leishmania* antibodies among a population of blood donors was reported at 15.6% {[21] #262}, indicating the presence of asymptomatic individuals among blood donors in different regions of the country.

Herein, our goal was to determine the prevalence of anti-*Leishmania* antibodies among blood donors of Salvador, Bahia, situated in northeastern Brazil. Although Salvador city *per se* is not endemic for VL, the HEMOBA/SESAB central blood bank draws donors from all over Bahia state and, as such, consists of a primordial sample for large-scale investigation. Of note, Bahia accounted for ~8% of VL cases registered in Brazil in 2012 and in 2013 {[22], #258}. We investigated both the presence of anti-*Leishmania* antibodies and of parasite DNA. For the latter, we employed a quantitative Real Time PCR (qPCR) {[23] #24}, targeting a single copy gene, and two conventional (end-point) PCR assays targeting: i) the Internal Transcribed Spacers present in rRNA {[24] #252} and ii) the conserved region of minicircle kDNA {[25] #253}.

## Methods

### Ethical considerations

This study was approved by the research ethics committee of CPqGM-FIOCRUZ-BA (No. 215/2010). Individuals involved in this study were required to sign the Informed Consent Form.

### Study design and population

The purpose of this study was to identify asymptomatic infection in blood donors attending the Hematology and Hemotherapy Foundation of Bahia State (HEMOBA/SESAB), situated in Salvador, BA, Brazil. The necessary sample size of approximately 220 individuals was estimated according to the following parameter: prevalence of an asymptomatic infection rate of 9%, based on a study conducted in Natal {[19] #37}, a VL endemic area {[26] #254}. Blood was obtained from 700 donors attending HEMOBA/SESAB and blood donations were screened over a period of nine months (January to September 2010). Donors resided in Salvador and other municipalities from BA.

### Sample collection

Whole blood was collected in quadruple blood packs (Fresenius/KAB), using the top-and-bottom system. Aliquots of 5 ml of peripheral blood were obtained following blood collection and were placed into Vacutainer^®^ tubes (Becton Dickinson) containing Heparin as anti-coagulant. Serum was separated by centrifugation and stored at -20°C for subsequent serologic studies. Aliquots of 200 μl of blood were used for DNA extraction.

### Soluble ***Leishmania***antigen

SLA was prepared from *L. infantum* MCAN/BR/00/BA262) and from *L. amazonensis* (MHOM/BR/1985/BA32) promastigotes maintained in Schneider (LGC Scientific) medium (Sigma) supplemented with 10% inactivated fetal bovine serum, 100 U/ml penicillin and 100 ug/ml streptomycin (Life Technologies). The parasites were initially submitted to 10 alternating cycles of freezing in liquid nitrogen and thawing in a water bath and were then centrifuged at 1600 × g, 4°C for 15 min. The supernatant containing SLA was collected and protein content was quantified using the Micro BCA TM Protein Reagent Kit assay (Pierce).

### ELISA to detect anti-***Leishmania***antibodies

Anti-*Leishmania* serology was performed by ELISA, as described {[27] #20}. Briefly, 96-well plates (Linbro/Titertek) were coated with 10 μg/mL of antigen (*L. infantum* or *L. amazonensis* SLA) and were incubated overnight at 4°C. After three washes with PBS/0.5% Tween, plates were blocked for 1 hour at 37°C with PBS/Tween 0.5% plus 1% fetal bovine serum (FBS) (Life Technologies). Plasma samples, diluted 1:100 in PBS/Tween 0.5% plus 1% FBS were added in duplicate and plates were incubated for 2 hours at room temperature. After three rounds of washing, wells were incubated with anti-human IgG conjugated to alkaline phosphatase (SIGMA) at a 1:2500 dilution in PBS/Tween 0.5% plus 1% FBS for 1 hour at room temperature. Plates were then washed and were further incubated for 30 minutes with a chromogenic solution of p-nitrophenyl phosphate (SIGMA) in sodium carbonate buffer pH 9.6 containing 1 mg/mL of MgCl_2._ In all experiments, the values obtained were subtracted from those obtained in the background. ELISA experiments were repeated twice yielding similar results. The ELISA cut-off value for *L. infantum* and *L. amazonensis* SLA was established using ROC curves (please see *Statistical analysis* below). In this case, we employed a panel of positive sera (*n* = 20), obtained from VL patients [defined by clinical and laboratory signs {[28] #280} and a positive bone marrow aspirate]. Control sera (*n* = 20) were obtained from negative individuals, living in areas free of *L. infantum*. These sera were randomly selected from a serum bank (LIP-CPqGM-FIOCRUZ).

### DNA extraction

DNA was extracted from 200 μL of whole blood using Illustra Genomic Prep Blood Mini Spin Kit™ (GE Healthcare), following the manufacturer’s instructions. DNA was eluted into 200 μL of Elution buffer and stored at -20°C. Purity of genetic material was assessed by absorbance at 260/280 nm.

### Real-Time PCR

Detection of *Leishmania* DNA by Real Time PCR was performed as described by {[23] #24}. Briefly, real time PCR was conducted in an ABI PRISM^®^ 7500 Sequence Detection System (Applied Biosystems) employing SYBR-Green^®^ PCR Master Mix (Invitrogen), 50 ng DNA and 200 nM of each forward and reverse primers. Thermal cycle conditions consisted of a denaturation step at 95°C for 10 minutes followed by 40 cycles at 95°C for 15 seconds and 60°C for 1 minute. Oligonucleotides (Fwd G6PD 5′-CCCGAGGGCAGCACTTG-3′ and Rev G6PD 3′-CCACCGGTCGTTGTTGATG-5′) were designed using PrimerExpress3.0 (Applied Biosystems) and were based on the *L. infantum* G6PD gene (glucose-6-phosphate dehydrogenase) (GeneBank: DQ212794.1). For parasite quantification, a standard curve was constructed based on DNA extracted from serial dilutions of *L. infantum,* ranging from 10^6^ to 10^1^ parasites. Analysis and acquisition of data were performed with SDS software (Applied Biosystems).

### Conventional PCR

End-point PCR targeting the 120-bp conserved region of the *Leishmania* kDNA minicircles {[25] #253} was performed as described {[29] #264}, employing primers 5′-GGG (G/T)AGGGGCGTTCT(G/C)CGAA-3′ and 5′-(G/C)(G/C)(G/C)(A/T)CTAT(A/T)TTACACCAACCC C-3′. The second end-point PCR assay targeted amplification of the segment containing ITS 1, 2 and the 5.8S region, within the rRNA gene locus {[24] #252}. Primers employed were IR1 (5′-GCTGTAGGTGAACCTGCAGCAGCTGGATCATT-3′) and IR2 (5′-GCGGGTAGTCCTGCCAAACACTCAGGTCTG-3′). Reactions were performed in a final volume of 20 μl, with 50 ng of DNA, 0.2 μM of each oligonucleotide and PCR supermix (Invitrogen), following manufacturer’s instructions. PCR conditions were as follows: denaturation at 94°C for 3 min, followed by 30 cycles of 94°C for 60 sec, 55°C for 60 sec and 94°C for 120 sec with a final extension of 72°C for 10 min. The amplification reactions were analyzed by agarose gel electrophoresis, followed by silver or ethidium bromide staining. DNA from the reference *L. infantum* strain was used as positive control for PCR reactions.

### Statistical analysis

Statistical analysis was performed using Prism v.5.0 (Graph Pad Software). ROC (Receiver Operating Characteristic) curves were calculated based on ELISA assays performed with SLA obtained from *L. amazonensis* and *L. infantum*. The cut-off, as determined by high sensitivity and specificity, from the highest probability of discrimination established by the curve, was therefore determined. The performance of each SLA was established by the parameters obtained from ROC curves values (AUC, *p* values and likelihood ratio). Non-parametric Mann–Whitney test was used to compare the frequency of seropositive individuals with a positive PCR result or a negative PCR result. The *p* value was considered significant when < 0.05. Agreement between PCR assays targeting *Leishmania* ITS and kDNA were determined by the *kappa* index.

## Results

### Anti-*L. infantum*serology in blood donors recruited at HEMOBA/SESAB

**Figure 1:**
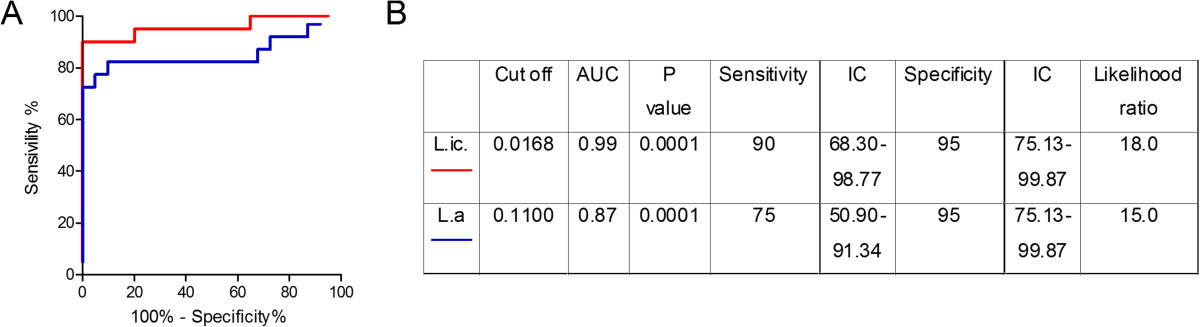
**ROC curve of antibody levels predict the positivity thresholds against SLA. (A)** ROC curves were constructed using data obtained in ELISA performed with SLA from *L. infantum* (L. i) or from *L. amazonensis* (L.a) and sera from confirmed VL patients (*n* = 20) and from control subjects from a non-endemic (*n* = 20) area. The red line represents the area under the curve for L.ic and the blue line represents the area under the curve for L.a. **(B)** Detailed information obtained for each ROC curve (cut-off values chosen, area under the curve, the values of *p*, and the sensitivity and specificity with a confidence interval of 95% and the likelihood ratio) is shown on the right.

Initially, we determined the sensitivity and specificity of the Soluble Leishmania Antigen (SLA) preparations [obtained from *L. infantum* or from *L. amazonensis* (the latter an etiological agent of Cutaneous Leishmaniasis)] when employed as the capture antigen in ELISA assays. With the results obtained, we constructed two ROC curves (Figure 1). *L. infantum* SLA performed better (AUC: 0.99, p < 0.0001, Likelihood ratio: 18.0) (Figure 1) when compared to *L. amazonensis* SLA (AUC 0.87; p < 0.0001 and Likelihood Ratio 15.0). Therefore, SLA from *L. infantum* presented higher sensitivity and specificity when compared to SLA from *L. amazonensis* and was selected for subsequent experiments. The cut off value, based on higher sensitivity and specificity of *L. infantum* SLA, was established as 0.01685.

### Cross-reaction among *Leishmania*and other blood borne pathogens

**Figure 2:**
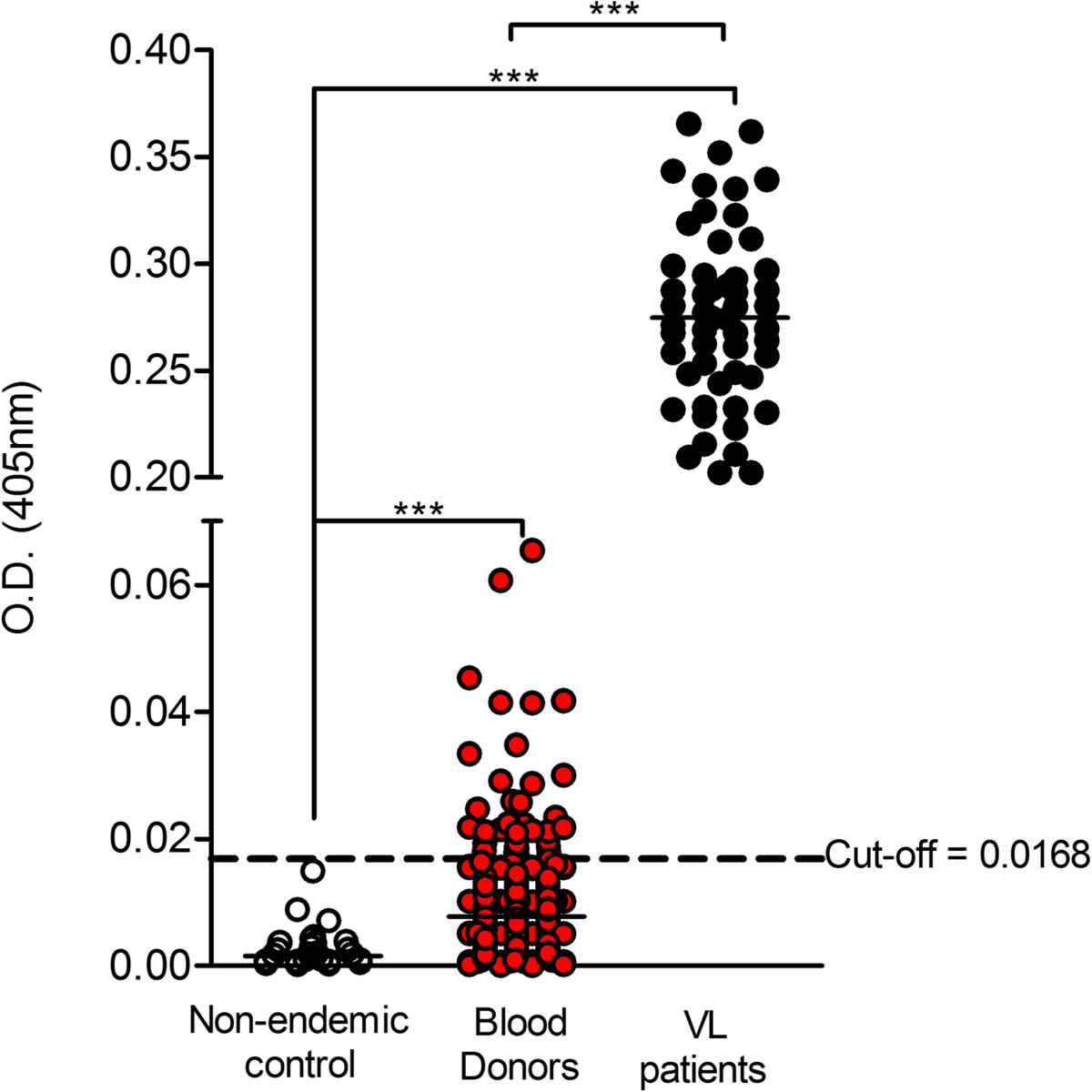
**Detection of Leishmania antibodies in blood donors of Salvador.** Samples from blood donors (*n* = 700) were tested for reactivity against *L. infantum* SLA by ELISA. Positive controls (*n* = 55) consisted of sera from individuals diagnosed with clinical VL and negative controls samples (*n* = 38) were obtained from volunteers living in a non-endemic area. All samples were tested in duplicate. ***p < 0.001.

Next, we investigated the presence of anti-*Leishmania* antibodies in the sera from 700 blood donors (74.5% male) attending HEMOBA/SESAB. The median age was 42 years: 31.7% individuals had between 19 and 28 years, 32.8% between 28 and 38 years, and 34% were older than 38 years of age. The reactivity of blood donors’ sera to *L. infantum* SLA was significantly higher compared to that obtained with negative controls (Figure 2). As expected, the reactivity of sera from confirmed VL patients was higher (p < 0.0001) compared to both blood donors and negative controls (Figure 2). Among the blood donors, we identified 38 individuals (5.4%) (81.5% male) that reacted above the established cut-off value (0.01685) and were thus considered seropositive against *L. infantum*. These 38 samples were also tested for cross-reactivity to other agents, following the routine blood screening investigation performed at HEMOBA/SESAB. Among the 38 *Leishmania* seropositive samples, 1/38 (0.02%) reacted positive for Chagas’ disease whereas another sample (1/38) reacted positively for Syphilis. Of note, among the 38 blood donors that were seropositive for *Leishmania*, 26/38 (68%) currently live in Salvador whereas 8/38 (21%) live in adjacent municipalities (address information was missing from four samples).

### Detection of *Leishmania*DNA in blood donors’ samples

**Figure 3:**
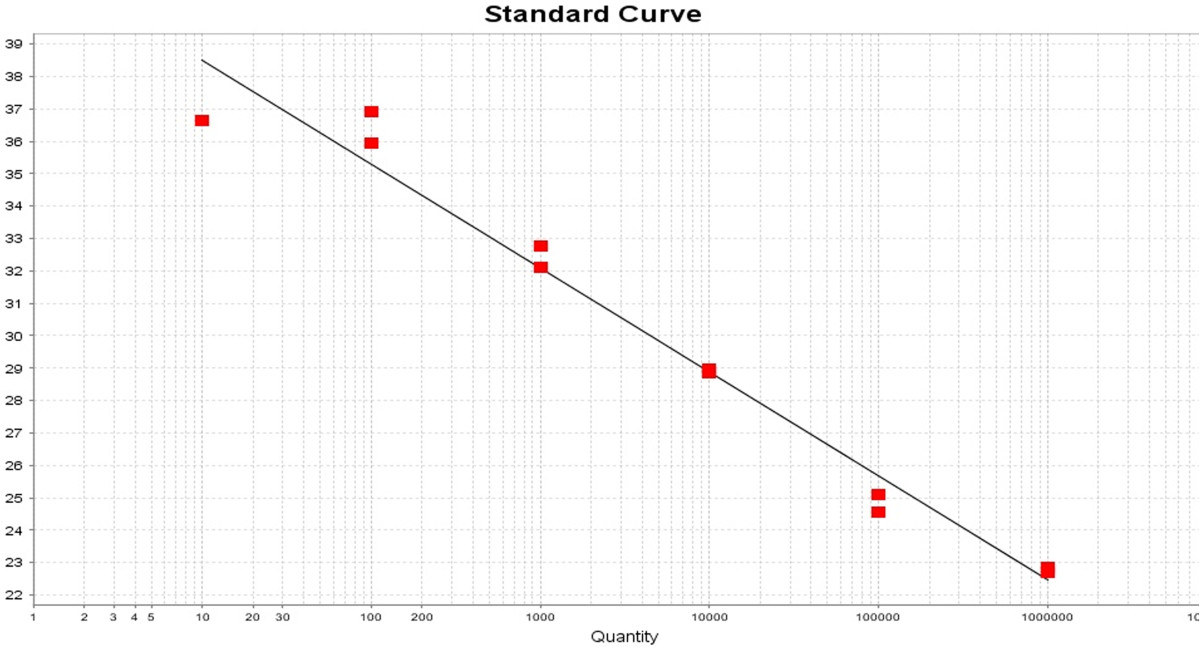
**Standard curve obtained with the G6PD** ***L. infantum*** **qPCR test.** The standard curve was generated by plotting the Ct against the input target quantity (in this case, serial dilutions of *Leishmania* DNA respresenting 10^6^ to 10^1^ parasites). Ct represents the fractional cycle number reflecting a positive PCR result differentiated from the background noise. Slope -3.206, PCR efficacy 105%, R^2^ = 0.971.

**Figure 4:**
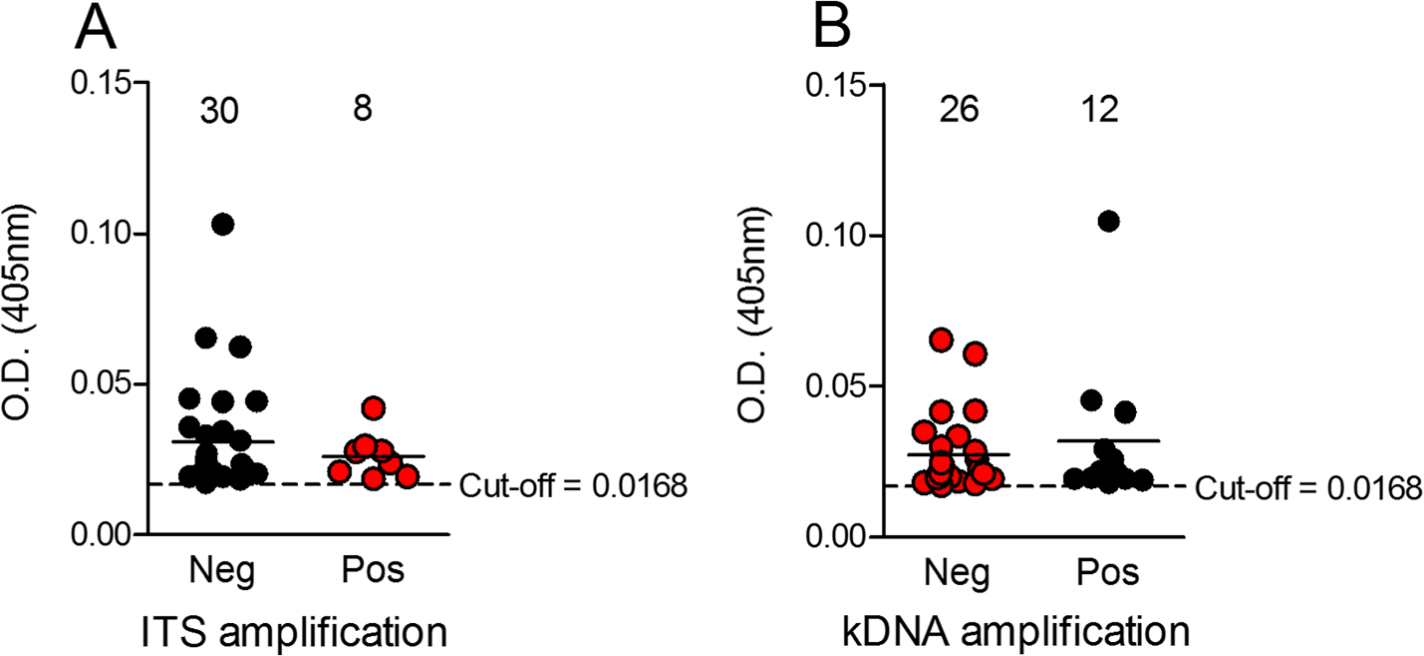
**Comparison of ELISA OD values obtained for blood donors with distinct PCR results.** Samples that were either negative or positive by ITS amplification **(A)** or kDNA amplification **(B)** were plotted against the respective OD values obtained by ELISA. Data are shown as the median OD obtained for each sample. Numbers above indicate the number of samples in each category (PCR negative or PCR positive).

We then investigated the parasite load in the 38 (5.4%) *Leishmania* seropositive samples (Figure 2). To do so, DNA extracted from the 38 blood samples was submitted to quantitative PCR (qPCR) targeting the single copy *G6PD* gene {[30] #243}. A standard curve (threshold cycle versus logarithm of the amplicon copy number) was constructed using DNA extracted from *L. infantum*, ranging 10^6^ to 10^1^ parasites (Figure 3). The standard curve showed a good linear correlation (R^2^ = 0.971) when plotted against the DNA equivalent to the number of parasites and the efficiency of the assay was 97%. We did not detect any level of interference due to the presence of human DNA as a similar efficiency of amplification was obtained when standard curves were generated from parasite DNA spiked with human DNA or from DNA extracted from a mixture of parasites plus human DNA (data not shown). Using the *G6PD* qPCR assay, we were not able to detect *Leishmania* DNA in any of the 38 blood donors that were seropositive by ELISA (Table 1). We then investigated whether end-point PCR assays targeting multiple copy genes would improve detection of parasite DNA. Targeting the region located between the small and the large subunit of the *Leishmania* rRNA gene array, including the Internal Transcribed Spacers (ITS) and the 5.8S rRNA {[24] #252}, we detected positive amplification in 8/38 (21.0%) samples (Table 1). In another assay, we amplified the conserved region of minicircles, present in 10,000-20,000 copies, located within the *Leishmania* kinetoplast {[25] #253}. This assay was positive in 26/38 (68.4%) samples (Table 1), raising the detection level by 47,4% when compared to ITS PCR (Table 1). The concordance between kDNA and ITS PCR was fair (kappa = 0.219) (Table 2). Lastly, positivity in either PCR assays, ITS or kDNA amplification, was dissociated from serology OD levels, determined by ELISA (Figure 4A and B, respectively).

**Table 1:** **Detection of Leishmania DNA in blood donors (n = 38) from Salvador, seropositive for** ***Leishmania***

PCR	G6PD {[30] #243}	ITS {[24] #252}	kDNA {[25] #253}
Positive, *n* (%)	0	8 (21.1)	26 (68.4)
Negative, *n* (%)	38 (100)	30 (78.9)	12 (31.6)
Total	38	38	38

**Table 2:** **Agreement between kDNA and ITS PCR results in blood donors (n = 38) with anti-** ***Leishmania*** **antibodies**

	kDNA		
ITS	Positive (%)	Negative (%)	Total
Positive, n (%)	8 (21)	0	8
Negative, n (%)	18 (47)	12 (31)	30
Total	26	12	38

*Kappa* = 0.219.

## Discussion

Asymptomatic *Leishmania infantum* infection can be detected initially with antibodies {[31] #275}; antibody responses decline with time in parallel to the development of a cell mediated immune response {[32] #282}. Therefore, serologic responses to *Leishmania* indicate acute infection, in the presence of symptoms or not. Reports of *Leishmania* transmission through the use of contaminated blood products {[14] #7; [10] #9} have raised the question whether asymptomatic infection among blood donors poses a problem for the blood supply, especially in regions where VL is endemic. Presently, we investigated the anti-*Leishmania* serologic response among blood donors from Salvador, northeastern Brazil. Additionally, we employed three PCR-based assays to determine the presence of *Leishmania* DNA in samples with positive serology.

Among the 700 blood donors recruited at HEMOBA/SESAB, the major blood donation center in Salvador, we detected 38 individuals (5.4%) with a positive serology to *Leishmania*. This finding is similar to that reported in Montes Claros (5.5%) {[33] #265} but lower than that observed in Natal (9%) {[20] #10} and in Campo Grande (15.6%) {[21] #262}, both of which are endemic regions for VL. The lower prevalence detected in our study may be due to the fact that Salvador is not a VL endemic area *per se*, differently from the other three municipalities. Additionally, differences in the sensitivity of the tests employed herein (SLA-based ELISA) versus those used in Natal (FML-ELISA) {[20] #10}, Montes Claros and Campo Grande (IFAT) {[33] #265; [21] #262} may contribute for the differing results. In our study, an ELISA assay employing *L. amazonensis* SLA was less sensitive when compared to *L. infantum* SLA (Figure 1), suggesting that the choice of antigen impacts on the results obtained. Among the 38 seropositive individuals detected herein, only one tested positive for Chagas disease under the routine screening performed at HEMOBA. We suggest that, in this current setting, routine screening for Chagas’ disease is not excluding *Leishmania* infection.

Molecular techniques have been successfully used to detect asymptomatic *Leishmania* infection {[34] #28; [26] #254; [35] #40; [36] #272} {[37] #273} and for the diagnosis and follow-up of VL patients {[38] #266; [39] #267}. Herein, we employed PCR to detect parasite DNA in the 38 individuals presenting anti-*Leishmania* antibodies. A quantitative assay, based on amplification of a single-copy target (*G6PD*) {[30] #243}, failed to amplify parasite DNA within the range established by the standard curve. This assay, however, was successful at identifying and estimating the number of parasites in biopsy samples from Cutaneous Leishmaniasis patients {[30] #243}. One possible reason can be the presence of very low levels of circulating parasites, hampering amplification of a single-copy gene. However, in a study conducted in Natal state, Brazil, positivity by qPCR (17%) was lower compared to positivity by ELISA (24.6%), even when a repetitive region was used as the amplification target {[26] #254}. In Minas Gerais state, Brazil, authors detected 56.5 parasites/ml of blood from seropositive individuals and this rate decreased to 7.8 parasites/ml after a one-year follow up, as measured by qPCR also targeting a repetitive region {[40] #270}. Indeed, a number of studies have reported differences in positivity by serological and molecular tests to detect *Leishmania* {[41] #31; [20] #10; [42] #255, [43] #13 [11] #250}. Herein, when we employed conventional end-point PCR targeting the ITSs within the rRNA gene array {[24] #252} or the conserved region within kDNA minicircles {[25] #253}, we were able to amplify *Leishmania* DNA in 8/38 (21%) and 28/38 (68%) seropositive samples, respectively, and agreement between both techniques was fair (*kappa* = 0.219). In a study with HIV/AIDS patients, prevalence of *Leishmania* based on ITS PCR was 1.8% {[44] #268} whereas by kDNA PCR, it increased to 11.8%, in France {[41] #31}, to 24% in Spain {[42] #255} and to 36.4% in Italy {[43] #14}. These results are in accordance with our observation that the higher the number of repetitions of a given amplification target, such as the ITSs or kDNA, the higher the sensitivity of the assay {[45] #278}. In our hands, kDNA amplification also proved a useful tool for the diagnosis of Cutaneous {[29] #264} and Mucocutaneous Leishmaniasis {[46] #281}. The fact that either ITS or kDNA amplification was negative in 12/38 samples (31.6%) with a positive *Leishmania* serology could again be explained by the presence of very low parasite numbers and, hence, few targets were available for PCR amplification. Another hypothesis is the presence of parasite remnants within phagocytic cells, rendering a positive amplification of *Leishmania* DNA by PCR.

In the current setting, blood screening for the presence of VL would decrease blood supply by 5%, negatively impacting on the blood supply provided by HEMOBA/SESAB. Hamsters transfused with blood from an experimentally infected animal developed disease as shown by presence of ascites, cachexia and death {[47] #44}. Foxhounds that received packed red blood cell transfusions from seropositive donors also tested positive for *Leishmania* antibodies {[48] #43}, indicating transmission of *Leishmania* to recipient dogs by blood transfusion. In a study conducted in Bangladesh, 1.195 blood donors were screened, antibodies were found in three individuals but none developed VL during a 6 month follow-up period {[11] #250}, suggesting that the number of circulating parasites is below the threshold necessary to initiate symptoms.

## Conclusions

Given that positive serology and/or presence of parasite DNA does not guarantee transfusional risk, the importance of our results lies in the recognition of the presence of cryptic *Leishmania* infection among blood donors from Salvador. *Leishmania* testing is not mandatory in blood banks in Brazil but knowledge of positive serology would impose blood elimination. Since VL is expanding and control measures are not efficient, a broader discussion is needed regarding the need to implement specific screening strategies and, possibly, the risk of *Leishmania* transmission by blood transfusion.

## Authors’ original submitted files for images

Below are the links to the authors’ original submitted files for images. Authors’ original file for figure 1 Authors’ original file for figure 2 Authors’ original file for figure 3 Authors’ original file for figure 4

## Abbreviations

ELISA: Enzyme-linked Immunosorbent Assay
IFAT: Immunofluorescence Antibody Test
ITS: Internal Transcribed Spacer
kDNA: Kinetoplastid DNA
PCR: Polymerase chain reaction
SLA: Soluble *Leishmania* Antigen
VL: Visceral Leishmaniasis.
