# Supplementary figures and images for: Serological survey of Leishmaniainfection in blood donors in Salvador, Northeastern Brazil
Source: BMC Infect Dis. 2014 Jul 30;14:422. doi: 10.1186/1471-2334-14-422 (PMC4122787; doi:10.1186/1471-2334-14-422)

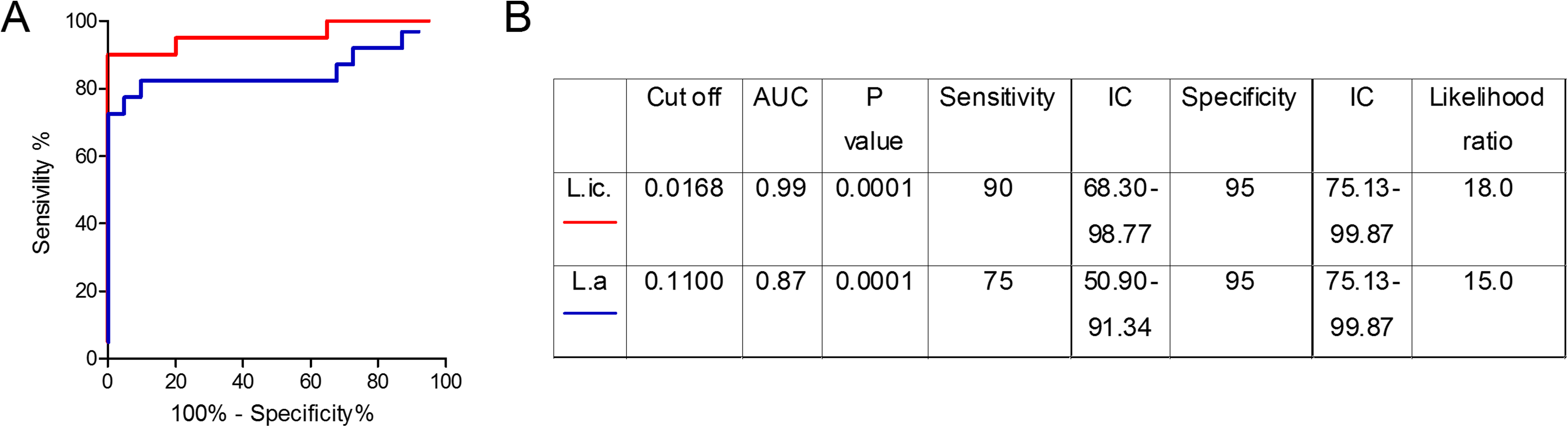

Supplement: Supplementary file 1 — Authors’ original file for figure 1 [file 12879_2014_3718_MOESM1_ESM.tif]

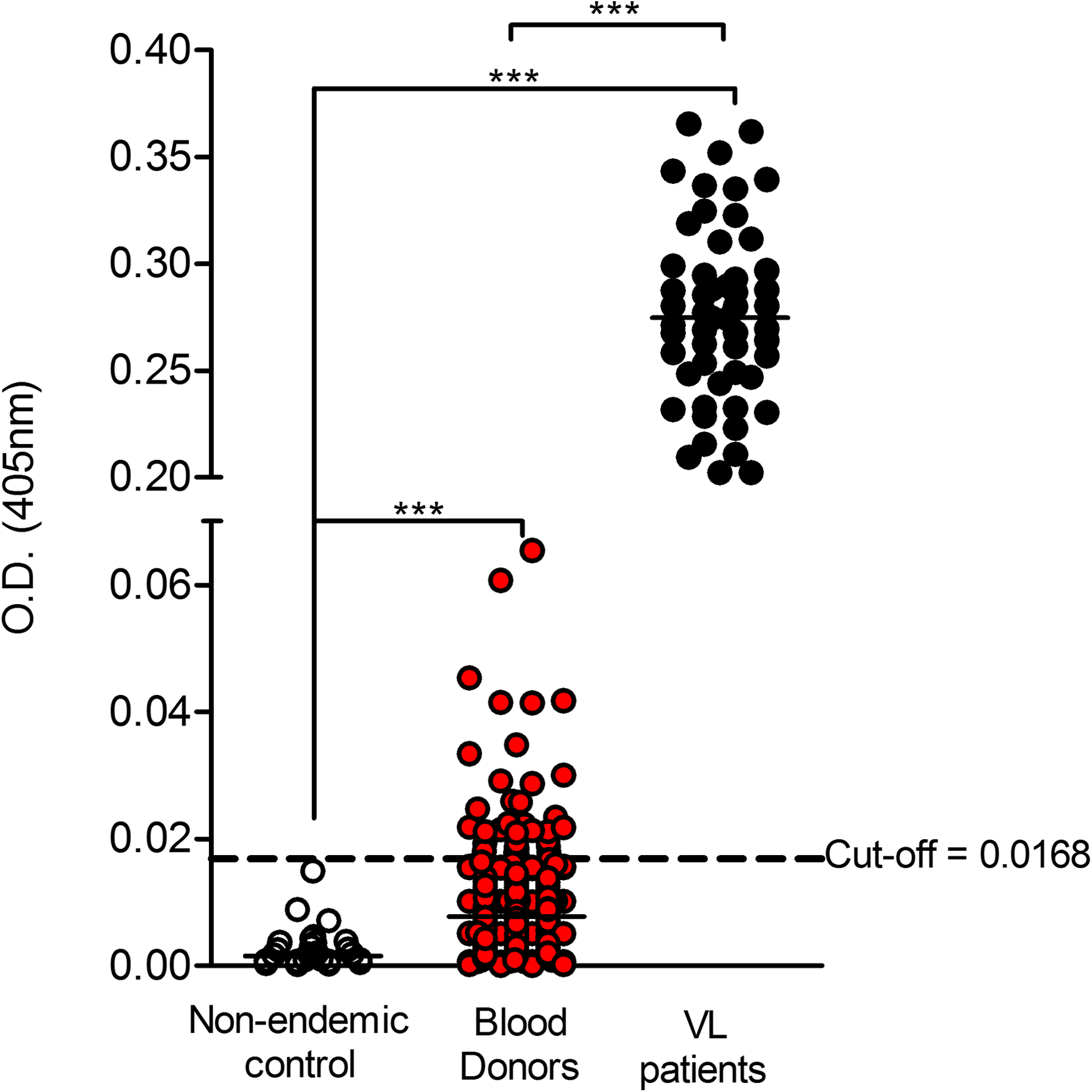

Supplement: Supplementary file 2 — Authors’ original file for figure 2 [file 12879_2014_3718_MOESM2_ESM.tif]

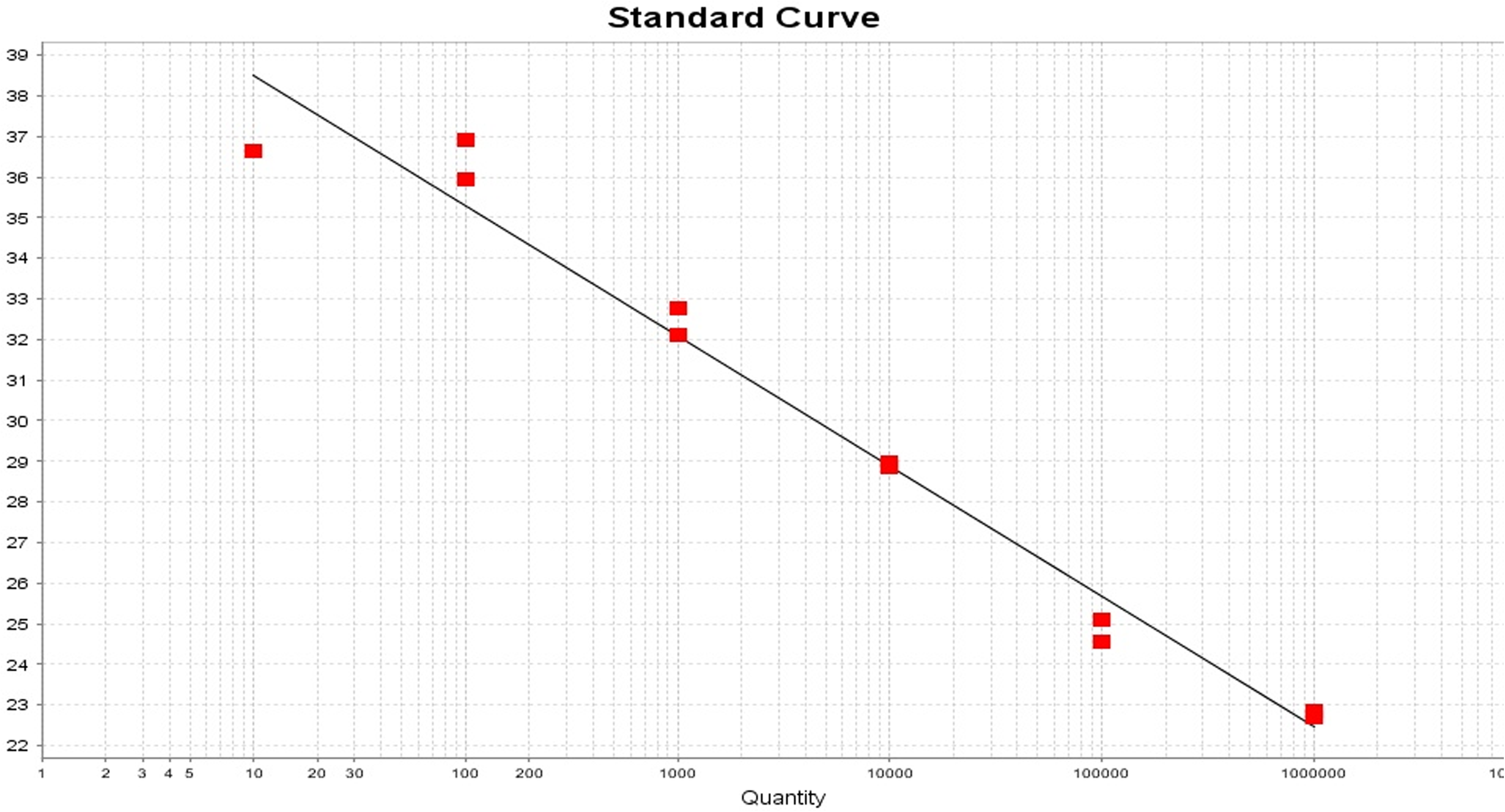

Supplement: Supplementary file 3 — Authors’ original file for figure 3 [file 12879_2014_3718_MOESM3_ESM.tif]

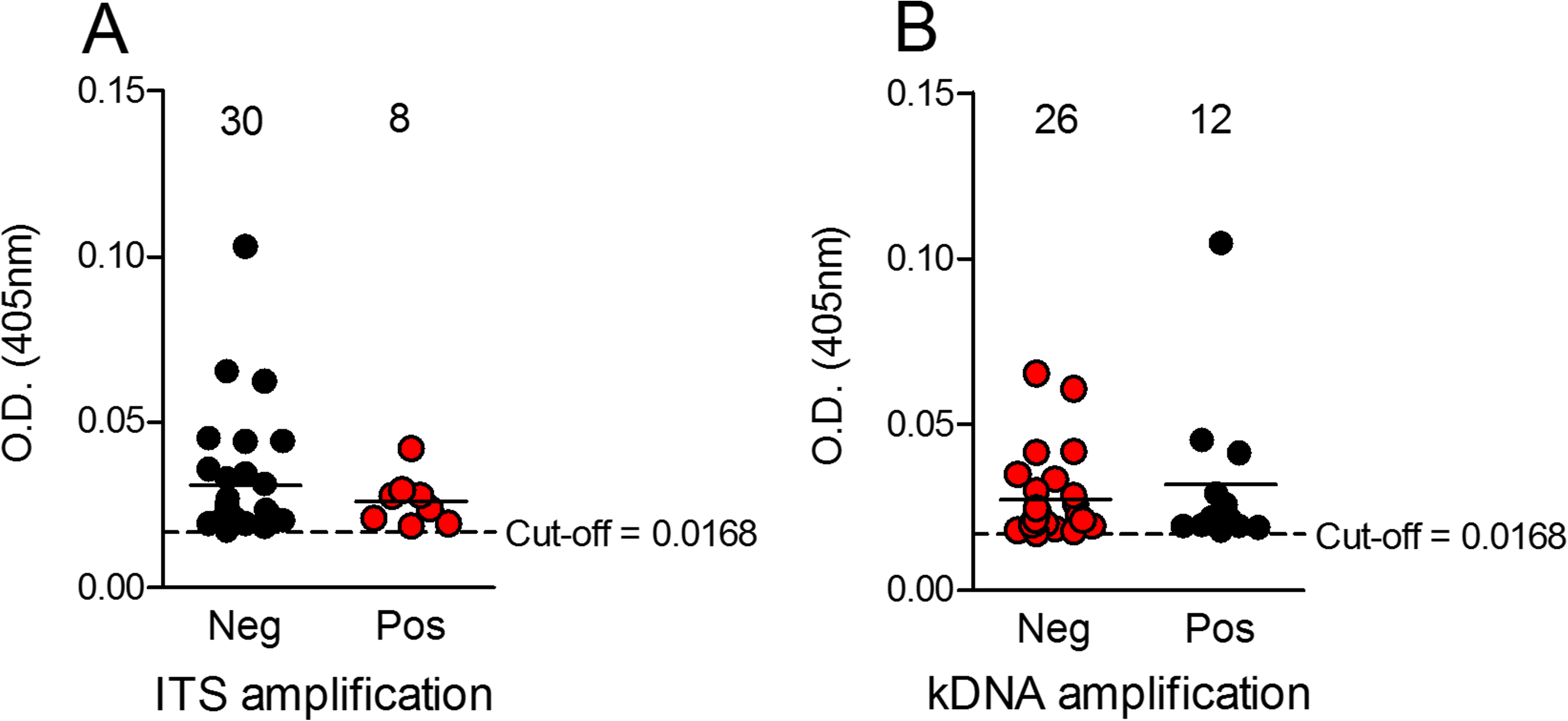

Supplement: Supplementary file 4 — Authors’ original file for figure 4 [file 12879_2014_3718_MOESM4_ESM.tif]
